# The HIV protease inhibitor Saquinavir attenuates sepsis-induced acute lung injury and promotes M2 macrophage polarization via targeting matrix metalloproteinase-9

**DOI:** 10.1038/s41419-020-03320-0

**Published:** 2021-01-11

**Authors:** Yao Tong, Zhuang Yu, Zhixia Chen, Renlingzi Zhang, Xibing Ding, Xiaohu Yang, Xiaoyin Niu, Mengzhu Li, Lingling Zhang, Timothy R. Billiar, Bruce R. Pitt, Quan Li

**Affiliations:** 1 Department of Anesthesiology, National Cancer Center/National Clinical Research Center for Cancer/Cancer Hospital and Shenzhen Hospital, Chinese Academy of Medical Sciences and Peking Union Medical College, 518116 Shenzhen, China; 2 Department of Anesthesiology, Ruijin Hospital, Shanghai Jiaotong University School of Medicine, 200000 Shanghai, China; 3 Department of Anesthesiology, Shanghai East Hospital, School of Medicine, Tongji University, 200120 Shanghai, China; 4 Department of Anesthesiology, Shanghai Tenth People’s Hospital, School of Medicine, Tongji University, 200072 Shanghai, China; 5 Department of Surgery, University of Pittsburgh School of Medicine, Pittsburgh, PA 15213 USA; 6 Department of Environmental and Occupational Health, University of Pittsburgh Graduate School Public Health, Pittsburgh, PA 15219 USA

**Keywords:** Inflammatory diseases, Inflammatory diseases

## Abstract

Imbalance of macrophage polarization plays an indispensable role in acute lung injury (ALI), which is considered as a promising target. Matrix metalloproteinase-9 (MMP-9) is expressed in the macrophage, and has a pivotal role in secreting inflammatory cytokines. We reported that saquinavir (SQV), a first-generation human immunodeficiency virus-protease inhibitor, restricted exaggerated inflammatory response. However, whether MMP-9 could regulate macrophage polarization and inhibit by SQV is still unknown. We focused on the important role of macrophage polarization in CLP (cecal ligation puncture)-mediated ALI and determined the ability of SQV to maintain M2 over M1 phenotype partially through the inhibition of MMP-9. We also performed a limited clinical study to determine if MMP-9 is a biomarker of sepsis. Lipopolysaccharide (LPS) increased MMP-9 expression and recombinant MMP-9 (rMMP-9) exacerbated LPS-mediated M1 switching. Small interfering RNA to MMP-9 inhibited LPS-mediated M1 phenotype and SQV inhibition of this switching was reversed with rMMP-9, suggesting an important role for MMP-9 in mediating LPS-induced M1 phenotype. MMP-9 messenger RNA levels in peripheral blood mononuclear cells of these 14 patients correlated with their clinical assessment. There was a significant dose-dependent decrease in mortality and ALI after CLP with SQV. SQV significantly inhibited LPS-mediated M1 phenotype and increased M2 phenotype in cultured RAW 264.7 and primary murine bone marrow-derived macrophages as well as lung macrophages from CLP-treated mice. This study supports an important role for MMP-9 in macrophage phenotypic switching and suggests that SQV-mediated inhibition of MMP-9 may be involved in suppressing ALI during systemic sepsis.

## Introduction

Sepsis remains a leading cause of death in the intensive care unit^1^, which has high rates of in-hospital mortality^2^. Amplified inflammatory response in the early phase of sepsis has been reported as a major cause of acute lung injury (ALI) that is characterized by increased vascular permeability, intrapulmonary retention of neutrophils, and synthesis of proinflammatory cytokines^3^. Macrophages play an indispensable role in ALI^4,5^ and phenotypic switching to M1 (classically activated macrophages that are proinflammatory and exert host defense against infection) over M2 (alternatively activated macrophages that are anti-inflammatory and associated with tissue remodeling) is a form of polarization associated with sepsis^6^. Therapy for sepsis remains primarily supportive and thus regulation of macrophage polarization may be a potential rational therapeutic target for mitigation of sepsis-induced ALI. Macrophages secrete various proinflammatory mediators, including interleukin-6 (IL-6), tumor necrosis factor-α (TNF-α), and matrix metalloproteinases (MMPs) following lipopolysaccharide (LPS) stimulation^7^. MMP-9 (or gelatinase B) belongs to a family of MMPs that are highly expressed in pathological processes including inflammation and tumor invasion^8,9^. MMP-9 is an inflammatory cytokine, and also acts as a regulator to promote the secretion of other cytokines by leukocytes^10^. The role of MMP-9 is somewhat unclear in ALI^11^ and may be specific to the cause of lung injury, the kinetics of underlying inflammation, concomitant changes in other MMP’s and/or the activity of endogenous inhibitors of MMP (e.g., tissue inhibitors of metalloproteinases (TIMPs)).

Human immunodeficiency virus (HIV) protease inhibitors (HIV-PIs), including saquinavir (SQV), lopinavir, and ritonavir, were used to interfere with virus reproduction for therapeutic intervention against HIV infection^12,13^. Studies have shown that HIV-PIs directly affect various signals, including tumor cell proliferation and survival, angiogenesis, antitumor immunity, and inflammation in HIV-free models^14–16^. We recently identified SQV in a medium-throughput screen and showed that SQV inhibited cathepsin V-mediated HMGB-MyD88-TLR4-induced TNF-α production in macrophages exposed to LPS^17,18^. The enhanced survivorship after cecal ligation puncture (CLP) extended previous observations by Weaver et al.,^19^ in which the mechanism of action of a combination of other HIV-PIs (nelfinavir and ritonavir) in decreasing CLP-induced mortality in mice was via inhibition of lymphocyte apoptosis. We chose to focus on another mechanism of action for a different HIV PI (SQV) related to its ability to inhibit MMPs in non-septic conditions. HIV-PIs prevent angiogenesis and cell invasion through their effects on the activity or production of MMPs^16,20^ and Barillari et al.^21^ found that SQV reduced MMP-9 expression and proteolytic activity in CaSki cells. Accordingly, we focused on the potential role of SQV as an MMP-9 inhibitor in preventing LPS-mediated M1 phenotypic switches in murine (transformed and primary) and human macrophages and mononuclear cells and in cells of intact mouse lung after CLP. In a circumscribed clinical study, we associated MMP-9 activity in peripheral blood mononuclear cells (PBMCs) from septic patients with the severity of their pathophysiological status.

## Results

### MMP-9 regulates M1 and M2 gene transcription in LPS-treated RAW 264.7 cells

**Fig. 1:**
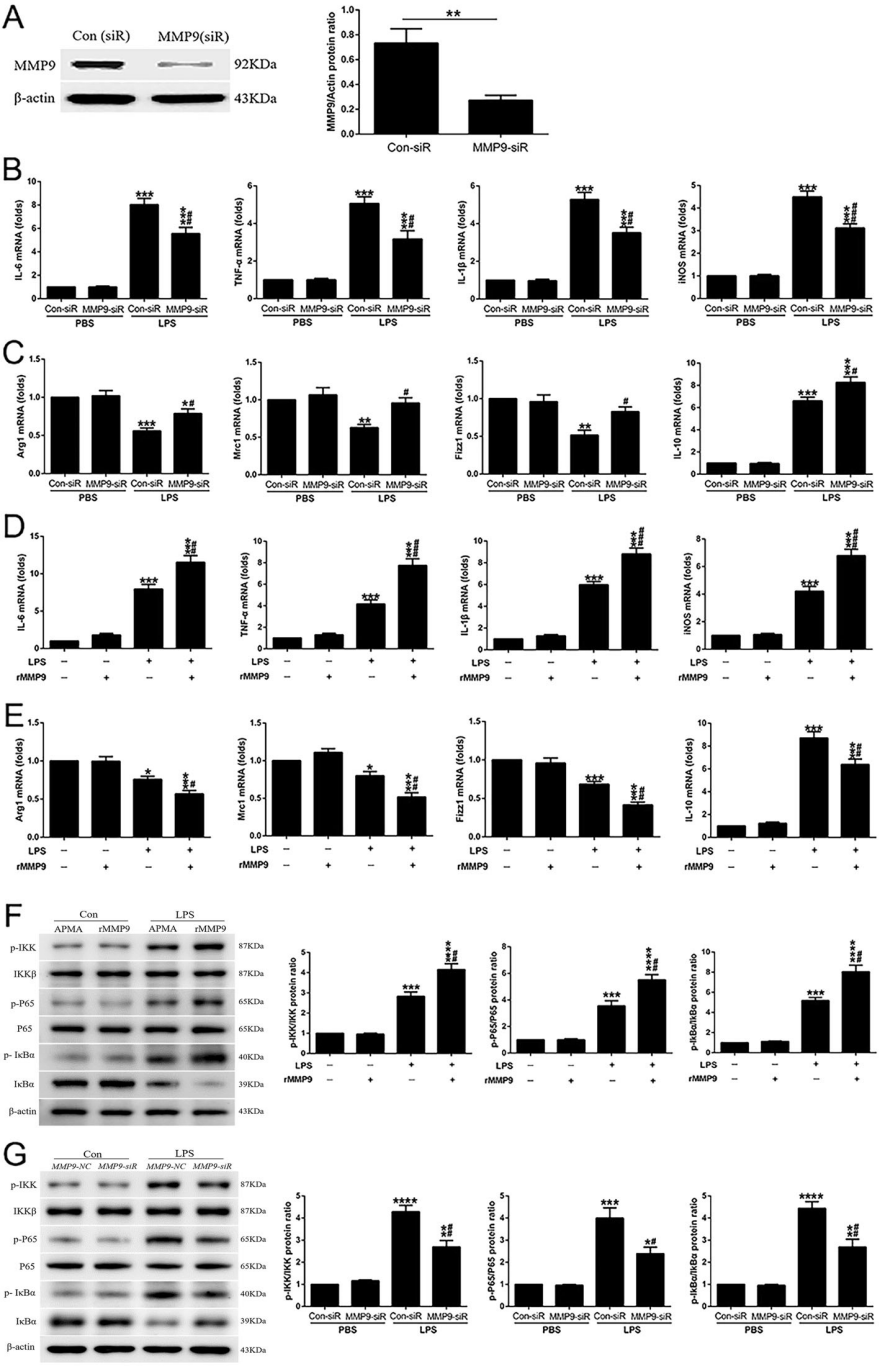
MMP-9 regulates M1 and M2 gene transcription in LPS-treated RAW 264.7 cells. **A** The transfection efficiency of *siMMP9* was assayed by western blot. **P < 0.01. **B**, **C** Expression of M1 and M2 marker genes was assessed in *MMP-9* siRNA- or *NC*-transfected RAW cells with or without LPS (100 ng/ml) challenge for 18 h. *P < 0.05, **P < 0.01, ***P < 0.001 versus PBS groups; ^#^*P* < 0.05, ^##^*P* < 0.01, ^###^*P* < 0.001 versus LPS (Con-siR) group. Expression of **D** M1- and **E** M2-associated genes was detected by quantitative PCR after 1 h of *p*-aminophenylmercuric acetate (APMA) or active rMMP-9 (5 ng/ml) pretreatment, followed by the presence or absence of LPS stimulation for 18 h. *P < 0.05, ***P < 0.001 versus LPS(−) groups; ^#^*P* < 0.05, ^##^*P* < 0.01, ^###^*P* < 0.001 versus LPS (APMA) group. **F** Raw cells were treated by APMA or active rMMP-9 (5 ng/ml) for 1 h prior to LPS challenged for 1 h, and then NF-κB signaling proteins (p-IKK, IKKβ, p-P65, P65, IκBα, and p-IκBα) were measured by western blot. ***P < 0.001, ****P < 0.0001 versus Con groups; ^##^*P* < 0.01 versus LPS (APMA) group. **G** Expression of NF-κB signaling proteins was assessed in *MMP-9* siRNA- or *NC*-transfected RAW cells with or without LPS (100 ng/ml) challenge for 1 h. All the results are from at least three independent experiments. *P < 0.05, **P < 0.01, ***P < 0.001, ****P < 0.0001 versus PBS groups; ^#^*P* < 0.05, ^##^*P* < 0.01 versus LPS (Con-siR) group. Data are represented as means ± SEM.

RAW 264.7 cells were transfected with small interfering RNA (siRNA) (or scrambled control) to *MMP-9* and exposed to LPS (100 ng/ml; 18 h). MMP-9 protein was in large part eliminated in the presence of siRNA to *MMP-9* (Fig. 1A). siRNA- to *MMP-9*-treated cells showed a significant decrease in LPS-mediated increases in M1 gene expression (interleukin-6 (*IL-6*), tumor necrosis factor-α (*TNF-α*), *IL-1β*, and inducible nitric oxide synthases (*iNOS*) (Fig. 1B) and significant increases in M2 gene expression (*Arg1*, *Mrc1*, *Fizz1*, and *IL-10*; Fig. 1C). In wild-type RAW 264.7 cells, recombinant MMP-9 (rMMP-9), by itself, did not affect markers of polarization, but in the presence of LPS, rMMP-9 increased the expression of M1 markers (messenger RNA (mRNA) for *IL-6*, *TNF-α*, *IL-1β*, and *iNOS*; Fig. 1D) and decreased LPS-induced changes in M2 markers (*Arg1*, *Mrc1*, *Fizz1* and *IL-10*; Fig. 1E).

Macrophage polarization can be regulated by nuclear factor-κB (NF-κB) signaling, which was stimulated to promote polarization of M1 macrophages and inhibited to promote polarization of M2 macrophages^22^. In addition, Liu et al.^23^ have found glutaminase-derived α-ketoglutarate (αKG) impairs the proinflammatory response of M1 macrophages by inhibiting the NF-κB pathway. Along this line, we determined to explore whether MMP-9 regulates macrophage polarization through the NF-κB signaling pathway. We found that rMMP-9, by itself, did not affect NF-κB signaling, but in the presence of LPS, rMMP-9 significantly increased phospho-NF-κB proteins, including p-IKK, p-P65, and p-IκBα in RAW 264.7 cells (Fig. 1F). siRNA to *MMP-9*-treated cells showed a significant decrease in LPS-mediated increases in phospho-NF-κB proteins (Fig. 1G). Moreover, we used PDTC (pyrrolidine dithiocarbamate ammonium) to inhibit NF-κB signaling to uncover the role of MMP-9 in the LPS-treated RAW cells. We found that PDTC-treated cells negated the effect of MMP-9 on promoting M1 macrophage polarization via detecting M1 biomarkers (Supplementary Fig. 1A–C). Collectively, these experiments suggest that MMP-9 has a contributory role in promoting LPS-mediated M1 polarization partially via NF-κB signaling in RAW 264.7 cells.

### SQV downregulates the expression and activity of MMP-9 in vitro and vivo studies

**Fig. 2:**
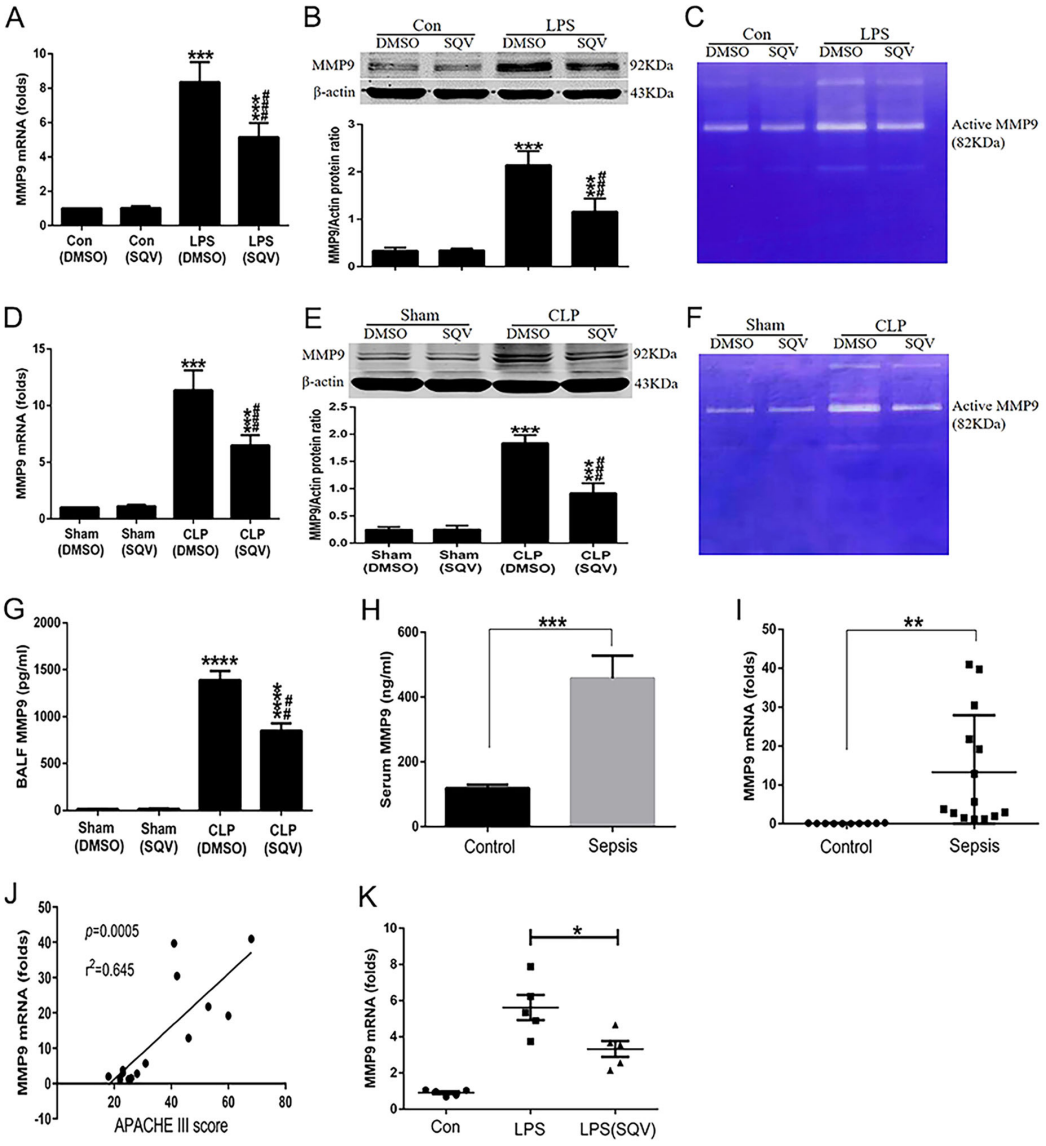
SQV downregulates the expression and activity of MMP-9 in vitro and vivo studies. RAW cells were administered with LPS or sterile PBS for 18 h following 1 h of SQV treatment, and then expression and activity of MMP-9 were detected by **A** quantitative PCR, **B** western blot, and **C** gelatin zymography. ***P < 0.001 versus Con groups; ^###^*P* < 0.001 versus LPS (DMSO) group. Mice were intraperitoneally administered SQV (10 mg/kg) or vehicle at 0 h (immediately) and 12 h after CLP. MMP-9 was assessed by **D** quantitative PCR, **E** western blot, **F** zymography in lung tissues, and **G** ELISA in BALF from four groups (*n* = 6 each group). ***P < 0.001, ****P < 0.0001 versus sham groups; ^##^*P* < 0.01, ^###^*P* < 0.001 versus CLP (DMSO) group. MMP-9 levels in the serum (**H**) and PBMCs (**I**) from septic patients and non-septic controls were detected by ELISA or qPCR. **P < 0.01, ***P < 0.001. **J** Correlation of MMP-9 expression and APACHE III scores in septic patients (*r*^2^ = 0.645, *P* = 0.0005). Data were analyzed by Spearman’s correlation test. **K** *MMP-9* mRNA levels were detected in PBMCs isolated from non-septic controls. *P < 0.05. Data are represented as means ± SEM.

As we have screened 5546 clinically used drugs and pharmacologically active compounds, we discovered that SQV has its new pharmacological role in inhibiting the excessive release of proinflammatory cytokines^17,18^. We intend to explore whether SQV could selectively or partially inhibit MMP-9 in the setting of inflammation. We first determined the effect of LPS on intracellular changes of MMP-9 in RAW 264.7 cells. LPS caused a significant increase in mRNA and cellular MMP-9 protein in RAW 264.7 cells (Fig. 2A, B). Further analysis by gelatin zymography in RAW 264.7 cells showed that MMP-9 enzyme activity was increased by LPS. SQV significantly inhibited the LPS-mediated increase in mRNA and protein of MMP-9 (Fig. 2A, B), as well as enzyme activity by gelatin zymography in RAW 264.7 cells (Fig. 2C).

In intact mice, we noted that CLP increased intrapulmonary *MMP-9* mRNA (Fig. 2D), protein (Fig. 2E), and enzyme activity (Fig. 2F). Bronchoalveolar lavage fluid (BALF) levels of MMP-9 in intact mice also increased after CLP (Fig. 2G). In all cases, when SQV was administered at the time of CLP and then again 12 h later, mRNA, protein, activity, and BALF MMP-9 levels were significantly less in CLP than after CLP and dimethyl sulfoxide (DMSO)-alone (control) treatment.

In a preliminary epidemiologic study, we noted that the expression of MMP-9 was greater in the serum (Fig. 2H) and PBMCs (Fig. 2I) of septic than non-septic patients. Moreover, the expression of MMP-9 was positively correlated with the severity of sepsis as measured by Acute Physiology, Age, and Chronic Health Evaluation III (APACHE III) scores (Fig. 2J). PBMCs from non-septic controls were pretreated with SQV for 1 h, followed by LPS stimulation for 18 h. We found that SQV significantly decreased *MMP-9* mRNA levels in LPS-challenged PBMCs (Fig. 2D).

### SQV inhibits LPS-mediated proinflammatory state and M1 polarization of RAW 264.7 cells

**Fig. 3:**
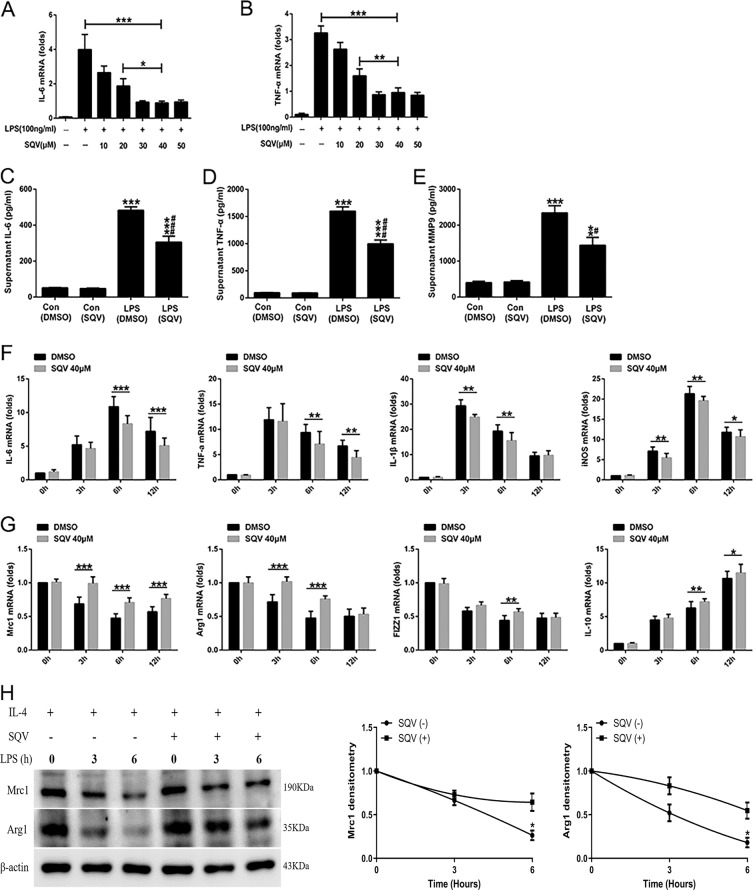
SQV inhibits LPS-mediated proinflammatory state and M1 polarization of RAW 264.7 cells. **A** *IL-6* and **B** *TNF-α* mRNA levels were detected in LPS-challenged RAW cells (6 h) after a dose course of SQV treatment for 1 h. *P < 0.05, **P < 0.01, ***P < 0.001. Secretion levels of **C** IL-6, **D** TNF-α, and **E** MMP-9 were assayed by ELISA in the supernatant of LPS-challenged RAW cells (18 h) following SQV treatment for 1 h. **P < 0.01, ***P < 0.001 versus Con groups; ^#^*P* < 0.05, ^###^*P* < 0.001 versus LPS (DMSO) group. RAW cells were pretreated with a dose course of SQV for 1 h, and then subjected to LPS stimulation for the indicated time points. Relative mRNA levels of **F** M1 and **G** M2 macrophages’ markers were measured by quantitative PCR. **H** RAW 264.7 cells were pretreated with IL-4 (10 ng/ml) for 24 h to induce M2 polarization, and then M2 macrophages were treated with SQV for 1 h prior to LPS stimulation at indicated times. Mrc1 and Arg1 protein expressions were measured by western blot. *P < 0.05, **P < 0.01, and ***P < 0.001. All the results are from at least three independent experiments. Data are represented as means ± SEM.

In preliminary experiments (data not shown), we noted that the peak increase in mRNA of *IL-6* and *TNF-α* occurred at 6 h after LPS was added to the medium of RAW 264.7 cells. We then noted that SQV dose-dependently inhibited this proinflammatory state (Fig. 3A, B). Besides, at 18 h post-LPS exposure, we noted a significant SQV-sensitive increase in IL-6 (Fig. 3C), TNF-α (Fig. 3D), and MMP-9 (Fig. 3E) in the medium of cultured RAW 264.7 cells.

LPS caused time-dependent increases in mRNA of M1 genes (*IL-6*, *TNF-α*, *IL-1β*, and *iNOS*) that were all partially but significantly inhibited in the presence of 40 µM SQV (Fig. 3F). Alternatively, LPS decreased the expression of mRNA of M2 genes (*Mrc1*, *Arg1*, *FIZZ1*) and this decrease was blunted by SQV consistent with the maintenance of M2 phenotype (Fig. 3G). Although LPS increased IL-10, SQV resulted in a further increase also consistent with M2 phenotype. Furthermore, RAW 264.7 cells were pretreated with IL-4 (10 ng/ml) for 24 h to induce M2 polarization, and then M2 macrophages were treated with SQV for 1 h prior to LPS stimulation at indicated times. We found that M2 markers were significantly reduced after LPS treatment, but this reduction was prevented by SQV (Fig. 3H).

### SQV inhibits LPS-mediated M1 macrophage polarization in primary bone marrow-derived macrophages and in primary macrophages isolated from intact mice after CLP

**Fig. 4:**
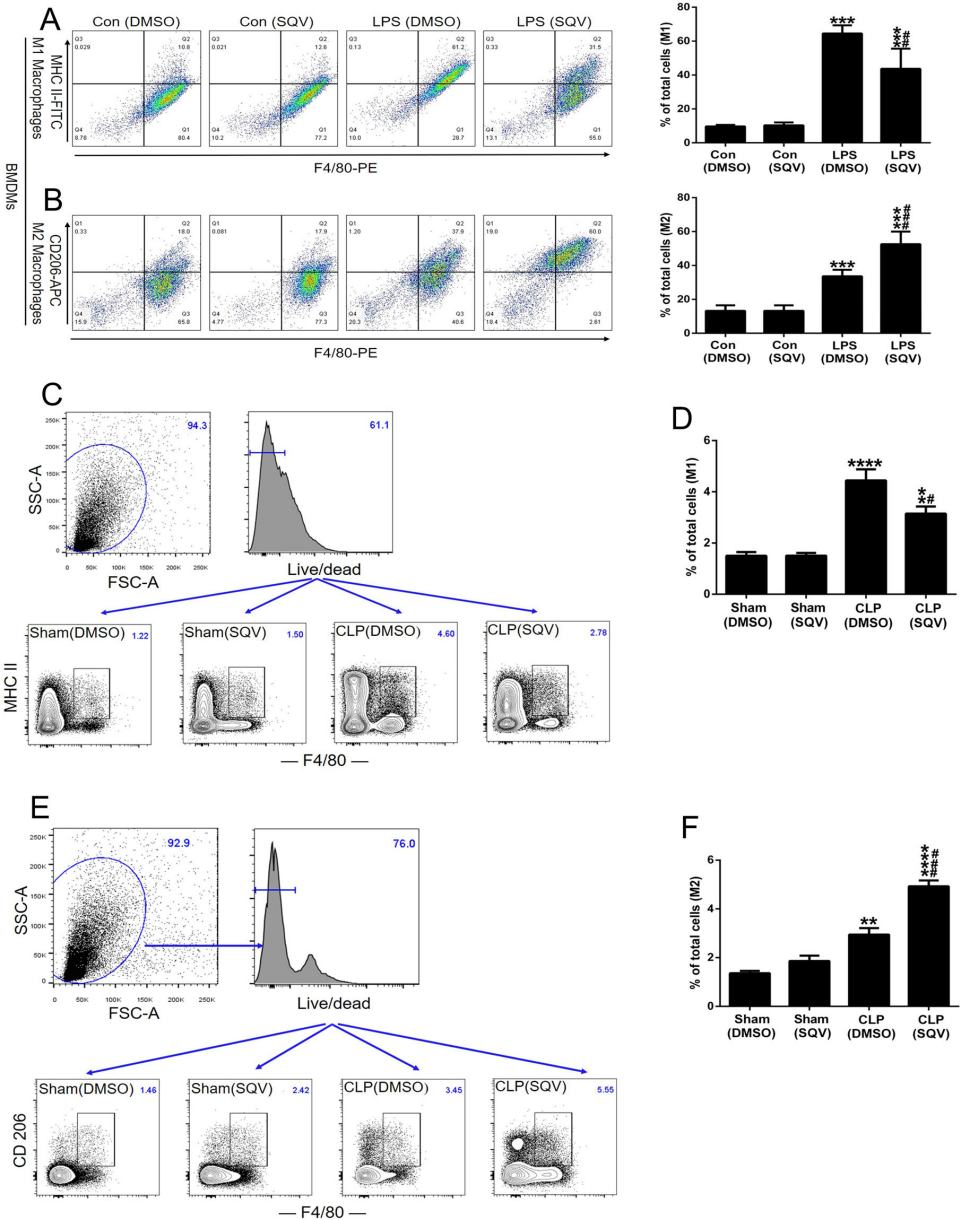
SQV inhibits LPS-mediated M1 macrophage polarization in primary bone marrow-derived macrophages and primary macrophages isolated from intact mice after CLP. BMDMs were incubated with SQV for 1 h, and then subjected to LPS stimulation for 18 h. Expression of **A** MHCII and **B** CD206 on BMDMs was detected by flow cytometry. ***P < 0.001 versus Con groups; ^##^*P* < 0.01, ^###^*P* < 0.001 versus LPS (DMSO) group. Representative histograms and cell percentages are depicted. Sham-operated mice and CLP-challenged mice were post-treated with or without SQV for 24 h, and then sacrificed to isolate total lung cells. Expression of **C**, **D** MHCII and **E**, **F** CD206 on lung macrophages was detected by flow cytometry. **P < 0.01, ****P < 0.0001 versus sham groups; ^#^*P* < 0.05, ^###^*P* < 0.001 versus CLP (DMSO) group. Representative histograms and cell percentages are depicted. All the results are from at least three independent experiments. Data are represented as means ± SEM.

We first determined whether SQV regulates polarization in cultured primary murine macrophages. Murine bone marrow-derived macrophages (BMDMs) were incubated with SQV for 1 h and then exposed to LPS. At 18 h, the expression of MHCII and CD206 was detected by flow cytometry (Fig. 4A). LPS caused an increase in the percentage of cells in M1 and M2, but the proportion of cells in the former was decreased and the proportion of cells in the latter increased with SQV pretreatment (Fig. 4B). Sham-operated mice and CLP-challenged mice were post-treated with (or without) SQV, and at 24 h, animals were killed and MHCII and CD206 expression on F4/80-PE-positive cells (i.e., macrophages) was determined by flow cytometry (Fig. 4C, D). SQV decreased the proportion of lung macrophages in M1 and increased the proportion in M2 (Fig. 4E, F). Thus, observations on the effect of SQV on polarization in transformed cells (e.g., RAW 264.7 cells; Fig. 3) were confirmed in isolated cultured primary murine macrophages (Fig. 4A, B) and short-term cultures of macrophages isolated from intact animals after CLP (Fig. 4C–F).

### SQV inhibits LPS-mediated M1 polarization of RAW 264.7 cells via MMP-9

**Fig. 5:**
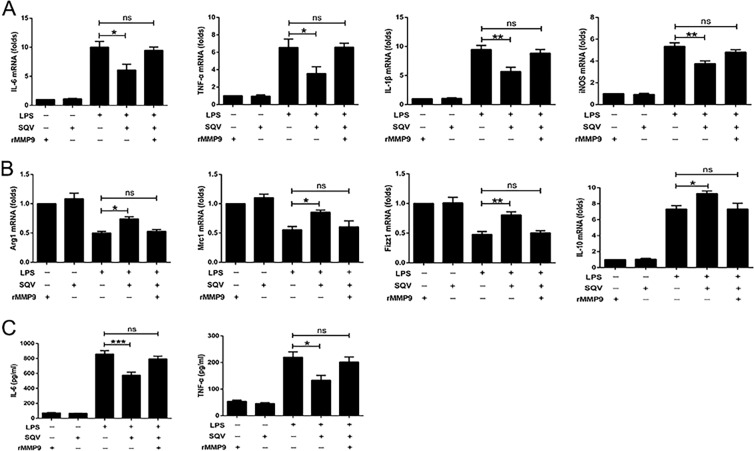
SQV inhibits LPS-mediated M1 polarization of RAW 264.7 cells via MMP-9. RAW cells were treated with rMMP-9 or SQV 1 h prior to 18 h of PBS/LPS challenged, and then expression of representative **A** M1, **B** M2 marker genes, and **C** the supernatant proinflammatory cytokines were assayed by quantitative PCR or ELISA. *P < 0.05, **P < 0.01, and ***P < 0.001. All the results are from at least three independent experiments. Data are represented as means ± SEM. *n.s*. Not significant.

After repeating our observations noted in Fig. 3 (LPS-mediated M1 polarization and cytokine production) and the partial reversal of LPS-mediated effects by SQV, we rescued the inhibition of SQV by exogenous rMMP-9 in RAW 264.7 cells (Fig. 5A–C). A similar phenomenon in which LPS-mediated M1 polarization was sensitive to SQV and the effects of SQV on M1 to M2 shifts in polarization could be reversed by exogenous rMMP-9 was noted in THP-1-derived macrophages (Supplementary Fig. 2A, B).

### SQV improves survival and attenuates lung and extrapulmonary injury in septic mice

**Fig. 6:**
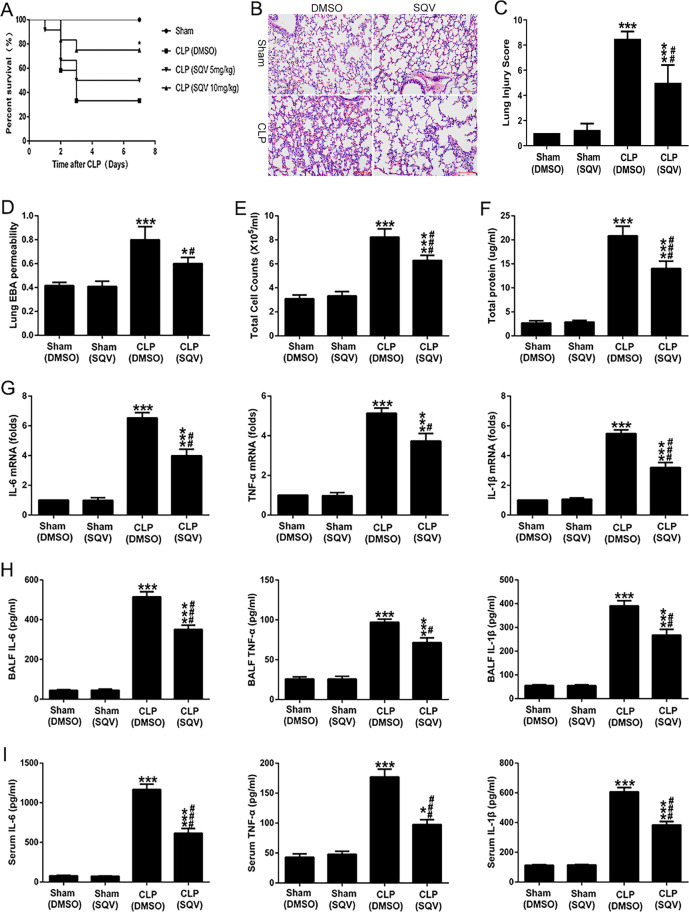
SQV improves survival and attenuates lung and extrapulmonary injury in septic mice. Mice were intraperitoneally administered SQV 5, 10 mg/kg or vehicle at 0 (immediately) and 12 h after CLP. **A** Survival rates (*n* = 12 per group) were detected. Data were analyzed by Mantel–Cox test (survival). *P < 0.05 versus CLP (DMSO) group. Mice were intraperitoneally administered SQV 10 mg/kg or vehicle at 0 (immediately) and 12 h after CLP. After 24 h, samples were collected. **B** Hematoxylin and eosin (H&E)-stained lung sections (×200 magnification), and **C** Lung injury score were measured. **D** Lung EBA permeability, **E** total cell counts, and **F** proteins in bronchoalveolar lavage fluid (BALF) were detected from mice. Proinflammatory cytokines from **G** lung tissues, **H** BALF, and **I** serum were measured from mice (24 h of sham or CLP operation) with or without SQV (10 mg/kg) treatment. *P < 0.05, ***P < 0.001 versus sham groups; ^#^*P* < 0.05, ^##^*P* < 0.01, ^###^*P* < 0.001 versus CLP (DMSO) group. Results in panels (**B**–**I**) are from four groups, each group has six mice. Data are represented as means ± SEM.

We first investigated whether SQV (5 and 10 mg/kg, intraperitoneal (i.p.)) could mitigate ALI when administered 12 h after CLP in intact mice (Fig. 6). There was a significant dose-dependent decrease in mortality after CLP with 5 and 10 mg/kg SQV (Fig. 6A) and a significant improvement in clinical assessment prior to any mortality (24 h post CLP) with 10 mg/kg SQV (data not shown). CLP resulted in histopathologic evidence of ALI in surviving mice 24 h after CLP (Fig. 6B) and SQV significantly decreased ALI as shown by lung injury score (Fig. 6C), alveolar capillary permeability (Fig. 6D), and cellular count (Fig. 6E) and total protein (Fig. 6F) recovered in BAL. Further evidence of CLP-induced ALI (and the mitigating effect of SQV) is shown in Fig. 6G–I. CLP caused a significant increase in *IL-6*, *TNF-α*, and *IL-1β* mRNA in the lung (Fig. 6G), BAL (Fig. 6H), and serum (Fig. 6I), respectively. SQV partially attenuated this increase in all compartments as shown in the last bar of each of the individual panels within Fig. 6C–I. In addition, CLP caused a significant increase in IL-6 and Arg1 proteins in the lung (Supplementary Fig. 3A–C), whereas SQV partially ameliorated IL-6 expressions and enhanced Arg1 levels.

CLP caused severe hepatocellular necrosis as detected by hematoxylin and eosin staining and light microscopy and this was partially inhibited by SQV as ascertained by liver injury score (Supplementary Fig. 4A). Liver injury was also apparent by CLP-mediated increases in serum levels of aspartate aminotransferase and alanine aminotransferase that also were partially inhibited by SQV (Supplementary Fig. 4B). Other indices of extrapulmonary injury with CLP, including renal dysfunction (i.e., elevated serum blood urea nitrogen and creatinine; Supplementary Fig. 4C) and systemic injury (i.e., elevated serum lactate and LDH; Supplementary Fig. 4D and E, respectively) were also sensitive to SQV. In this part of our study, SQV (10 mg/kg) was administered at the beginning of laparotomy and again at 12 h after CLP.

## Discussion

In the current study, we showed that SQV could reduce mortality and mitigate ALI (Fig. 6) and hepatocellular necrosis (Supplementary Fig. 4) secondary to polymicrobial sepsis in intact mice. SQV significantly inhibited M1 phenotype and increased M2 phenotype in LPS exposed RAW 264.7 (Fig. 3) and primary murine BMDMs (Fig. 4A, B), as well as lung macrophages from CLP-treated mice (Fig. 4C–F). LPS increased MMP-9 expression (mRNA, protein, and enzyme activity via gelatin zymography) in in vitro and in vivo studies. rMMP-9 exacerbated LPS-mediated M1 switching and siRNA to *MMP-9* inhibited LPS-mediated M1 phenotype (Fig. 1). SQV inhibition of this switching was reversed with rMMP-9 (Fig. 5 and Supplementary Fig. 2), suggesting an important role for MMP-9 in mediating LPS-induced M1 phenotype. MMP-9 was significantly elevated in the serum of 14 septic patients compared to 10 non-septic controls and *MMP-9* mRNA levels in PBMCs of these 14 patients correlated with their clinical (e.g., APACHE III) assessment (Fig. 2H–K).

### MMP-9 in ALI due to systemic sepsis

MMPs are a large family of proteins that regulate degradation and formation of the extracellular matrix and participate in the inflammatory response^24^. We focused on MMP (and in particular MMP-9 or gelatinase B) as a candidate contributor to macrophage phenotypic switching in ALI since the alveolar basement membrane is extensively remodeled in acute respiratory distress syndrome (ARDS)^11^, and in the acute phase of ARDS, MMP-9 levels have been noted in BAL fluid^25,26^. Nonetheless, the role of MMP-9 is somewhat unclear in ALI and may be specific to the cause of lung injury, the kinetics of underlying inflammation, concomitant changes in other MMP’s, and/or the activity of endogenous inhibitors of MMP (e.g., TIMPs). We noted that BALF MMP-9 levels were enhanced in septic mice and intrapulmonary *MMP-9* mRNA, protein, and enzyme activity increased after CLP. This finding is in line with other studies proposing that circulating levels of MMP-9 are increased in inflammatory injury models^27,28^, although there is some discrepancy regarding findings in the serum^29^ and BAL fluid^30^ in human ARDS. In this regard, we did note increased serum levels of MMP-9 from septic patients as described by others in human sepsis^28,31^. Nonetheless, studies have shown that serum levels of MMP-9 were not correlated with mortality in septic patients^28,32^. We collected PBMCs from septic patients to detect the relationship between *MMP-9* gene expression with the severity of sepsis in patients and found *MMP-9* mRNA levels have a positive correlation with APACHE III score.

MMP-9-null mice have worse outcomes in sterile forms of lung injury, including ventilator-induced lung injury^33^ and ozone induced lung inflammation^34^, suggesting that MMP-9 is protective. In contrast, MMP-9-null mice are resistant to sterile injury induced by immunoglobulin G complexes^35^ and CMT3 (an MMP-9 inhibitor) protects rats against VILI^36^. Relevant to the current study, MMP-9-null mice are resistant to CLP^28^ and COL3 (a putative MMP-9 inhibitor) protects rats and pigs against ALI from CLP^37^ and LPS^38^, respectively. Many other studies have suggested that MMP-9 may be injurious in ALI by using either somewhat nonspecific MMP-9 inhibitors or reporting associations with protection and decreased MMP-9 activity against H2S^39^, H3N2 influenza^40^, and LPS^41^. Thus, it appears, at least in the case of CLP, that MMP-9 contributes to ALI secondary to systemic sepsis. Since MMP-9 zymogen can affect M1- and M2-polarized macrophages in angiogenesis^42^ and macrophage polarization is potentially important in ALI, we further pursued the relationship of MMP-9, macrophage phenotype, and the potential for SQV to exert its therapeutic effect by inhibiting this pathway.

### A role for MMP-9 in macrophage phenotypic switching in ALI

The ability of macrophages to differentiate along a continuum from M1 (classically activate/inflammatory) to M2 (alternatively activated/regenerative) underlies their indispensable role in inflammatory disease including ALI^32,43^. We noted enhancement of M1 macrophages (and suppression of M2 phenotype) in RAW 264.7 and primary murine BMDMs as well as lung macrophages from CLP-treated mice. Tipping this balance away from M1 towards M2 phenotype may have therapeutic potential in ALI as Herold et al.^44^ demonstrated that M2-polarized exudate macrophages decreased alveolar epithelial cell injury and enhanced lung barrier function in *IL-1ra*^−/−^ monocytes.

By using a simple model of LPS-treated macrophages and cytokine production and M1/M2 switching, it was possible to more securely identify a role of MMP-9. Although rMMP-9 itself did not affect cytokine production or phenotypic switching, it did enhance such effects by LPS. LPS, itself, increased cytokine production and expression of MMP-9 in RAW and human THP-1 monocytes and increased M1 and decreased M2 phenotype in RAW cells and BMDMs. Most importantly, siRNA to *MMP-9* inhibited this phenotypic switch to M1 and rMMP-9 restored the switch to M1 after LPS in the presence of SQV (see below). Accordingly, a component of LPS-mediated switching can be confidently ascribed to MMP-9.

### SQV, ALI, M1/M2 switching, and MMP-9

Although SQV was first used in clinical practice as an HIV protease inhibitor in 1995^12^, it subsequently has been shown to act as an antineoplastic agent as well as regulating inflammation^18,45^. We reported that in a medium-throughput screen of 5546 Food Drug Administration-approved drugs, first-generation HIV-PIs were effective agents against HMGB-1-induced TNF-α production in RAW246.7 cells^17^. We^18^ subsequently showed that SQV inhibited disulfide HMGB-1-mediated TNFα production in isolated human monocyte-derived macrophages and freshly isolated mouse peritoneal macrophages by inhibiting cathepsin V (or cathepsin L, murine homolog) necessary for TLR4-MyD88^46^ activation. In model systems of HMGB-1–TLR4 axis, we^18^ noted that SQV increased the survival of intact mice after CLP or minimized liver damage after warm ischemia–reperfusion. We^47^ noted further evidence for a role for SQV in affecting sterile injury (intimal hyperplasia after mouse carotid artery wire injury) involving HMGB-1 and MyD88–TLR4 axis, cathepsin L, and monocyte recruitment^48^. Collectively, these studies point to a potential therapeutic role for SQV in septic and sterile injuries involving monocytic TLR4-mediated inflammation and led logically to the current study.

In this study, we showed that SQV improved survival and ameliorated ALI in septic mice. Although we used a dose of 10 mg/kg SQV to post-treat mice at the time of CLP, and 12 h thereafter, Pribis et al.^18^ have displayed a relatively low dose for 3-day survivorship starting at 24 h following CLP. Thus, SQV has the potential to mitigate injury, an important consideration for ultimate clinical utility. We showed that SQV markedly decreased the expression and activity of MMP-9 in lung tissues from septic mice. We noted that SQV dose-dependently inhibited LPS-mediated increases in *IL-6* and *TNF-α* mRNA in RAW cells and decreased synthesis and release of these cytokines and MMP-9 into the culture medium. SQV promoted M2 phenotype in LPS-treated RAW cells and primary murine macrophages, as well as macrophages isolated from CLP-treated mice. The inhibitory effect of SQV was reversed by the addition of rMMP-9 in RAW cells strongly linking the mechanism of action via SQV’s ability to inhibit MMP-9 activation. Accordingly, the current study extends original observations on other HIV-PIs (nelfinavir and ritonavir) by Weaver et al.^19^ that showed protection against CLP via inhibition of lymphocyte apoptosis.

## Conclusions

Collectively, in intact mice and human subjects and transformed and primary murine and human mononuclear cells, this comprehensive study supports an important role for MMP-9 in macrophage phenotypic switching and suggests that SQV-mediated inhibition of MMP-9 may contribute to the efficacy of SQV in inhibiting ALI in systemic sepsis.

## Materials and methods

### Human samples

The clinical study was conducted at the Shanghai Tenth People’s hospital. The Human Research Committees from the Shanghai Tenth People’s hospital approved the study, and informed consent was obtained from each participant or immediate family member. Fourteen patients were identified as sepsis prospectively according to the definitions of the Sepsis-related Organ Failure Assessment 3.0. The characteristics of our patient population are summarized in Table 1. Serum or PBMCs from septic patients and those from healthy volunteers were performed to detect MMP-9 protein by enzyme-linked immunosorbent assay (ELISA) and MMP-9 mRNA by standard quantitative PCR, respectively. We have registered our clinical trial in the Chinese Clinical Trial Registry (Number: ChiCTR-ROC-17011095; Date: 8 April 2017; URL: http://www.chictr.org.cn).

**Table 1:** Characteristics of patients/healthy volunteers.

Characteristics	Non-sepsis (*n* = 10)	Sepsis (*n* = 14)
Age, years	58.50 ± 2.75	59.93 ± 1.95
Male, *n* (%)	7 (70)	10 (71.43)
MMP-9 (ng/ml)	119.0 ± 10.71	457.6 ± 70.40
APACHE III score	–	36.14 ± 4.20
SOFA score	–	6.71 ± 0.53
Etiology, *n* (%)		
Pneumonia	–	6 (42.86)
Peritonitis	–	3 (21.43)
Multiple injuries	–	2 (14.28)
Urosepsis	–	2 (14.28)
Other	–	1 (7.14)
Comorbidities, *n*		
None	9	3
Respiratory	0	6
Cardiovascular	0	9
Diabetes	1	6
Chronic renal disease	0	2
Ventilatory support, *n* (%)	–	3 (21.43)
30-day mortality, *n* (%)	0 (0)	2 (14.28)

Data are expressed as the mean ± SEM or number (%).

*APACHE III* Acute Physiology, Age, Chronic Health Evaluation III, *SOFA* Sequential Organ Failure Assessment.

### Reagents

SQV was obtained from Dr. Yousef Al-Abed (The Feinstein Institute for Medical Research, Manhasset, NY). PDTC was purchased from MedChemExpress. Recombinant mouse MMP-9 (R&D, 909-MM) and human MMP-9 (R&D, 911-MP) was activated according to the standard protocol. APMA (*p*-Aminophenylmercuric acetate) was purchased from Sigma-Aldrich (A9563). PMA (phorbol 12-myristate-13-acetate; Sigma-Aldrich 79346) and LPS (055:B5) were obtained from Sigma-Aldrich. The fluorescein-conjugated monoclonal antibodies (F4/80, MHCII, and CD206) and the isotype controls were purchased from BD Pharmingen (San Diego, CA). Recombinant murine macrophage colony-stimulating factor (M-CSF) (315-02) and IL-4 (214-14) were purchased from PeproTech.

### Cell culture

RAW 264.7 cells were from ATCC (TIB-71) and cultured in Dulbecco’s modified Eagle’s medium with 10% fetal bovine serum. THP-1 cells were from ATCC (TIB-202). PBMCs and THP-1 cells were cultured in RPMI-1640 medium (Gibco) with 10% fetal bovine serum. Penicillin (50 μg/ml) and streptomycin (50 μg/ml) were added to cell cultures. All the obtained cell lines were authenticated and tested for mycoplasma contamination before conducting experiments.

### Animals

Male wild-type mice (C57BL/6; 8–12 weeks) were bought from Shanghai Laboratory Animal Co. Ltd (SLAC, Shanghai, China). All mice were fed in a laminar-flow, specific pathogen-free atmosphere at the Shanghai Tongji University. Mice in this study were randomly assigned to four groups by an investigator who was blinded to the group allocation. Animal protocols were approved by the Ethics Committee of the University of Tongji and the experiments were performed in accordance with the National Institutes of Health Guidelines for the Use of Laboratory Animals.

### Sepsis induced by CLP

Mice were anesthetized by i.p. administration of 100 mg/kg ketamine and 10 mg/kg xylazine. After the abdominal fur was shaved, a 2 cm midline incision was made through the skin and peritoneum. The cecum was then isolated and ligated with a 4-0 silk ligature at 75% the distance between the distal pole and the base of the cecum. Cecal puncture (“through-and-through”) was initiated at the mesentery by a 21 G needle and proceeded in the antimesenteric direction after ligation. The cecum was then returned to the peritoneal cavity and the abdominal incision was closed with 4-0 sterile synthetic absorbable suture.

### Alveolar capillary permeability by Evans Blue albumin (EBA)

Alveolar capillary permeability was estimated with EBA based on our previous description^49^. EBA was administered through the vena jugularis externa 1 h before sacrificing all models, and then the lung tissues were reserved to do further research.

### Measurement of cytokines

According to the manufacturer’s instructions, circulating or BALF levels of TNF-α, IL-6, MMP-9, and IL-1β were determined by ELISA (RayBiotech).

### RNA extraction, reverse transcription PCR, and quantitative PCR

Total RNA was extracted from cells or lung tissues using TRIzol reagent (Life Technologies, Grand Island, NY) and 1 μg total RNA was reversed into complementary DNA (cDNA) using First-strand Cdna Synthesis Kit (Takara) according to standard protocols. Quantitative PCR (qPCR) was performed in triplicate on a LightCycler 480 Instrument II machine (Roche Life Science) using SYBR Green PCR mixture (Kapa Biosystems). Primers for qPCR are displayed in Supplementary Tables 1 and 2.

### Western blot analysis

Western blotting for MMP-9 and NF-κB pathway proteins, Mrc1 and Arg1, was performed as a standard protocol. Membranes were blocked with 5% skimmed milk, incubated with primary antibody against MMP-9 (ABclonal, A2095 for THP-1 cells; Abcam, ab38898 for RAW cells and lung tissues from mice), NF-κB proteins (CST, NF-κB Pathway Sampler Kit #9936), Mrc1 (ABclonal, A8301), and Arg1 (ABclonal, A1847) overnight. Membranes were washed and incubated at room temperature for 2 h with secondary antibodies (1:2000). Then, membranes were washed three times and pictures were taken using the Odyssey Infrared Imaging System (LI-COR).

### Bone marrow- and THP-1-derived macrophages

For BMC purification, femurs and tibias were taken from killed mice and flushed with a 1 ml syringe filled with phosphate-buffered saline (PBS) containing 0.1% bovine serum albumin and 20 mM HEPES (pH 7.4). Following RBC lysis, the BM cell suspensions were filtered through a 40-µm cell strainer (Falcon). Cells were stimulated with complete medium containing M-CSF (20 ng/ml) for 6-7 days to acquire macrophages.

THP-1, a promonocytic cell line derived from a human acute monocytic leukemia patient, cultured at 37 °C with 5% CO_2_ in RPMI-1640 medium containing 10% fetal bovine serum, 2 mM glutamine, 50 μg/ml penicillin, and 50 μg/ml streptomycin. THP-1 cells (1 × 10^6^/ml) were stimulated with 50 ng/ml PMA for 3 days to obtain THP-1-derived macrophages.

### Cell staining for flow cytometry

BMDMs and total lung cells from mice were labeled with the fluorochrome-conjugated primary antibodies to F4/80, MHCII, and CD206 for 30 min. For cell death analysis, cells were digested with 0.05% Trypsin-EDTA (Gibco,Thermo Fisher Scientific), washed with PBS, and then co-stained with Annexin V/PI (V13242, Invitrogen) followed by flow cytometry. Cells were gated on F4/80- and MHCII-positive expression, which were identified as M1 macrophages. Cells were gated on F4/80- and CD206-positive expressions, which were identified as M2 macrophages. Unstained and fluorescein-conjugated isotypic cells were used as controls. Samples were acquired on a flow cytometry analyzer (LSR II; BD Biosciences), and data were analyzed with the DIVA software (BD Biosciences).

### Immunohistochemical staining

Lung sections were performed in 4% paraformaldehyde overnight, and then embedded and cut into 5 μm, which were placed onto slides. These slides were heated at 67 °C for 30 min and dewaxed in dimethylbenzene. Slides were then dehydrated in a concentration gradient of alcohol and pretreated with microwave heat-induced epitope retrieval. After that, slides were incubated with the primary antibody of IL-6 (1:200; ABclonal) or Arg1 (1:100; ABclonal) for 24 h at 4 °C and then the secondary antibody at 1:50 dilution was applied for 1 h at 28 °C. Slides were stained by diaminobenzidine and then visualized using a digital camera (Olympus) combined with a light microscope at ×200 magnification.

### Knockdown *MMP-9* gene expression by lentiviral siRNA vector

Lentiviral siRNA vector was used as previously described to knockdown *MMP-9* gene expression^50^. Briefly, *MMP-9* siRNA targeting sequence or control siRNA sequence was synthesized and inserted into a lentiviral siRNA expression vector. The recombinant lentiviral siRNA vector particles were packaged in 293 T packaging cell lines. Cell culture media were then collected and titers of the lentiviral vectors (multiplicity of infection) were determined as described. Lenti-MMP-9 siRNA (titer 3.0E + 8 TU/ml) or lenti-MMP-9-NC (titer 1.6 E + 8 TU/ml) was used to infect Raw cells by directly adding to culture media. At 4–8 h after infection, the infection media were replaced with fresh media. Efficiency of lentiviral vectors infection was monitored under fluorescent microscopy by the lentiviral vector co-expressed green fluorescent protein (GFP). More than 80% of Raw cells were positive for GFP. Also, knockdown of *MMP-9* gene expression was determined by western blot as shown in Fig. 1A.

### Gelatin zymography assay

Samples were settled by lysis buffer and centrifuged at 12,000 × *g* for 15 min at 4 °C. Supernatants were extracted and mixed with PBS. Then, they were mixed in a sample buffer containing 62.5 mmol/l Tris-HCl (pH 6.8), 10% glycerol, 2% sodium dodecyl sulfate, and 0.00625% (w/v) bromophenol blue without being boiled. Mixtures were loaded in separating gel containing 0.1% (w/v) gelatin. Electrophoresis was started at a constant voltage of 120 V. After electrophoresis, the gel was soaked in 0.25% Triton X-100 two times (30 min/time) at room temperature and rinsed in deionized water. The gel was then incubated at 37 °C for 20–40 h in the incubation buffer containing 50 mmol/l Tris-HCl (pH 7.6), 20 mmol/l NaCl, 5 mmol/ CaCl_2_, and 0.02% NaN_2_. The gel was then stained in 0.25% (w/v) Coomassie blue R-250 (30% methanol and 10% acetic acid) for 15–30 min, then de-stained in the same solution without the Coomassie brilliant blue dye, pour destaining solution, and kept until the clear bands are visible against a blue background. The clear zone in the blue field reflects the gelatinolytic activity.

### Statistical analysis

Results in this study are expressed as means ± SEM of independent experiments. Group comparisons were performed using *t* test or one-way analysis of variance (ANOVA) with Tukey’s post hoc test. Survival curve was measured by Mantel–Cox test. Correlation analysis was detected by Spearman’s correlation test. *P* < 0.05 (*P < 0.05, **P < 0.01, and ***P < 0.001) was considered statistically significant. All statistical analyses were carried out using the GraphPad Prism 6.0 program.

## Supplementary information

Supplementary Figure Legends

Supplementary Figure 1

Supplementary Figure 2

Supplementary Figure 3

Supplementary Figure 4

Supplementary Table 1

Supplementary Table 2
