# Supplementary material for: The HIV protease inhibitor Saquinavir attenuates sepsis-induced acute lung injury and promotes M2 macrophage polarization via targeting matrix metalloproteinase-9
Source: Cell Death Dis. 2021 Jan 11;12(1):67. doi: 10.1038/s41419-020-03320-0 (PMC7798387; doi:10.1038/s41419-020-03320-0)
Supplement: Supplementary file 1 — Supplementary Figure Legends [file 41419_2020_3320_MOESM1_ESM.docx]

**SUPPLEMENTARY FIGURE LEGENDS**

**Supplementary Figure 1.** PDTC rescued rMMP9 effects on promoting M1 marker genes’ expression in LPS challenged RAW 264.7 cells. RAW cells were pre-treated with rMMP-9 and/or PDTC (50 μM) 1 h followed by 18 h of PBS/LPS challenged, and then expressions of representative M1 marker genes (A-C) were assayed. ^*^*P*＜0.05, ^**^*P*＜0.01. All the results are from at least three independent experiments; Data represent means ±SEM.

**Supplementary Figure 2.** MMP-9 partially regulates macrophage polarization in THP-1 derived macrophages following LPS treatment. THP-1 derived macrophages were pre-treated with rMMP-9 and/or SQV 1 h followed by 18 h of PBS/LPS challenged, and then expressions of representative (A) M1 and (B) M2 marker genes were assayed. ^*^*P*＜0.05, ^**^*P*＜0.01, ^***^*P*＜0.001. All the results are from at least three independent experiments; Data represent means ±SEM.

**Supplementary Figure 3.** SQV regulates expressions of macrophage markers in the lung of septic mice. Mice were intraperitoneally administered SQV 10 mg/kg or vehicle at 0 (immediately) and 12 h after CLP. 24 h later, samples were collected. (A) Western results and (B and C) IHC staining of IL-6 or Arg1 were showed in the lung from four groups. ^*^*P*＜0.05, ^**^*P*＜0.01, ^***^*P*＜0.001 versus sham groups; ^#^*P*＜0.05, ^##^*P*＜0.01 versus CLP (DMSO) group. All the results are from at least three independent experiments; Data represent means ±SEM.

**Supplementary Figure 4.** SQV ameliorates sepsis-induced multi injury in mice. (A) Hematoxylin and eosin (H&E)–stained liver sections from four groups. (B) Serum ALT and AST levels were measured for hepatocellular injury. Serum levels of (C) BUN, creatinine, (D) lactate and (E) LDH were detected 24 h after CLP challenged. ^*^*P*＜0.05, ^**^*P*＜0.01, ^***^*P*＜0.001 versus sham groups; ^#^*P*＜0.05, ^##^*P*＜0.01, ^###^*P*＜0.001 versus CLP (DMSO) group. All the results are from at least three independent experiments; Data represent means ±SEM.
