# Supplementary figures and images for: The HIV protease inhibitor Saquinavir attenuates sepsis-induced acute lung injury and promotes M2 macrophage polarization via targeting matrix metalloproteinase-9
Source: Cell Death Dis. 2021 Jan 11;12(1):67. doi: 10.1038/s41419-020-03320-0 (PMC7798387; doi:10.1038/s41419-020-03320-0)

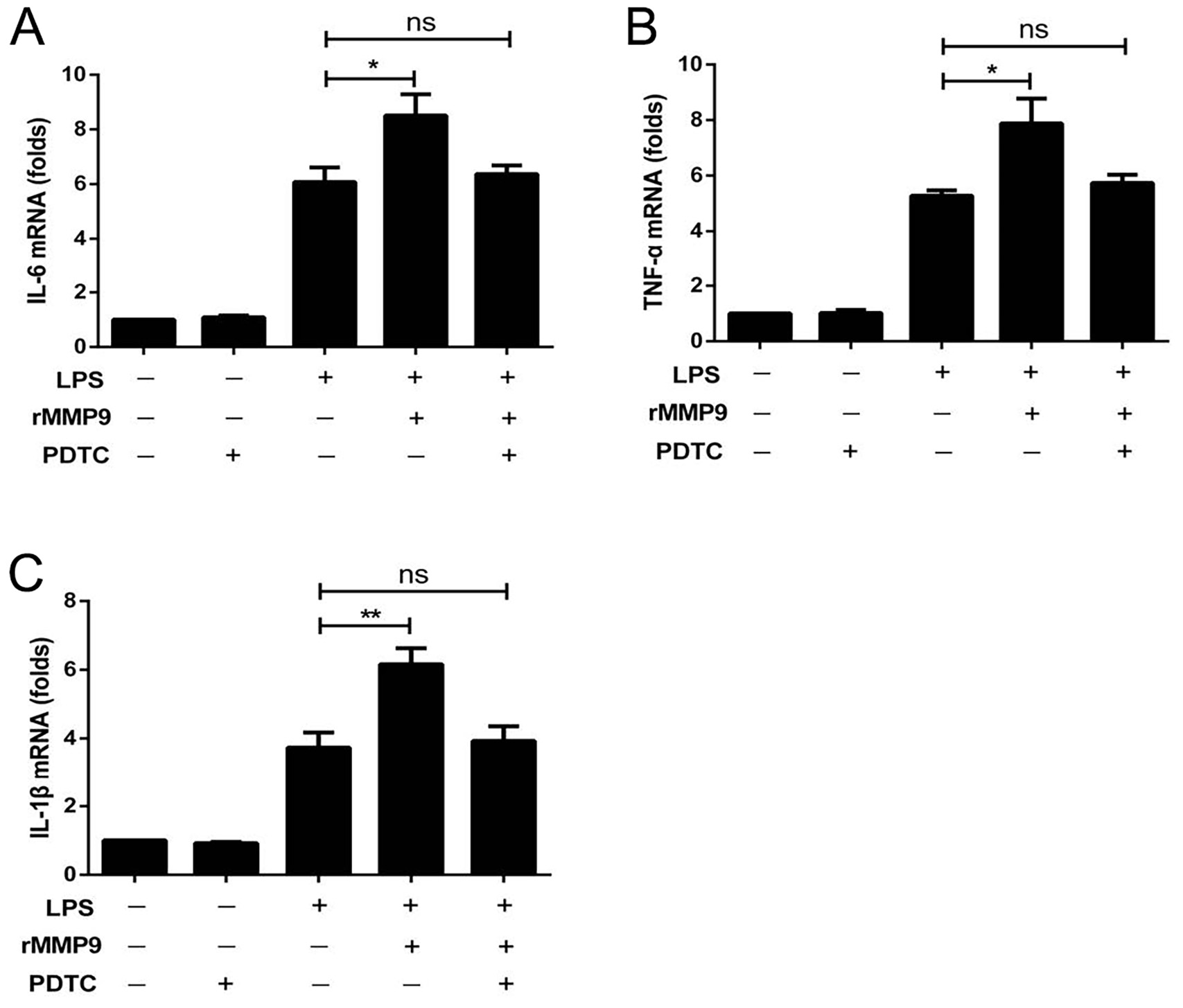

Supplement: Supplementary file 2 — Supplementary Figure 1 [file 41419_2020_3320_MOESM2_ESM.tif]

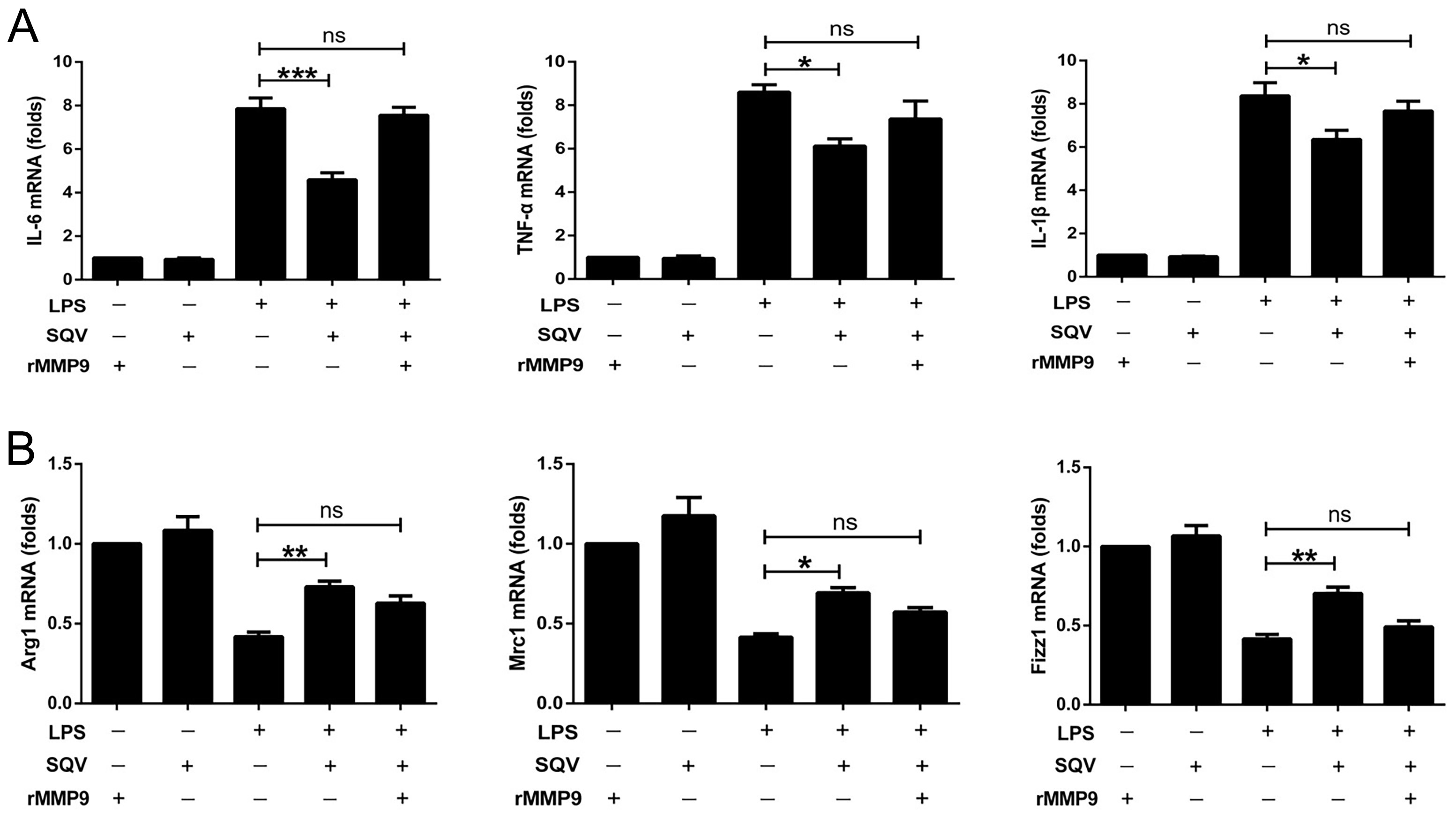

Supplement: Supplementary file 3 — Supplementary Figure 2 [file 41419_2020_3320_MOESM3_ESM.tif]

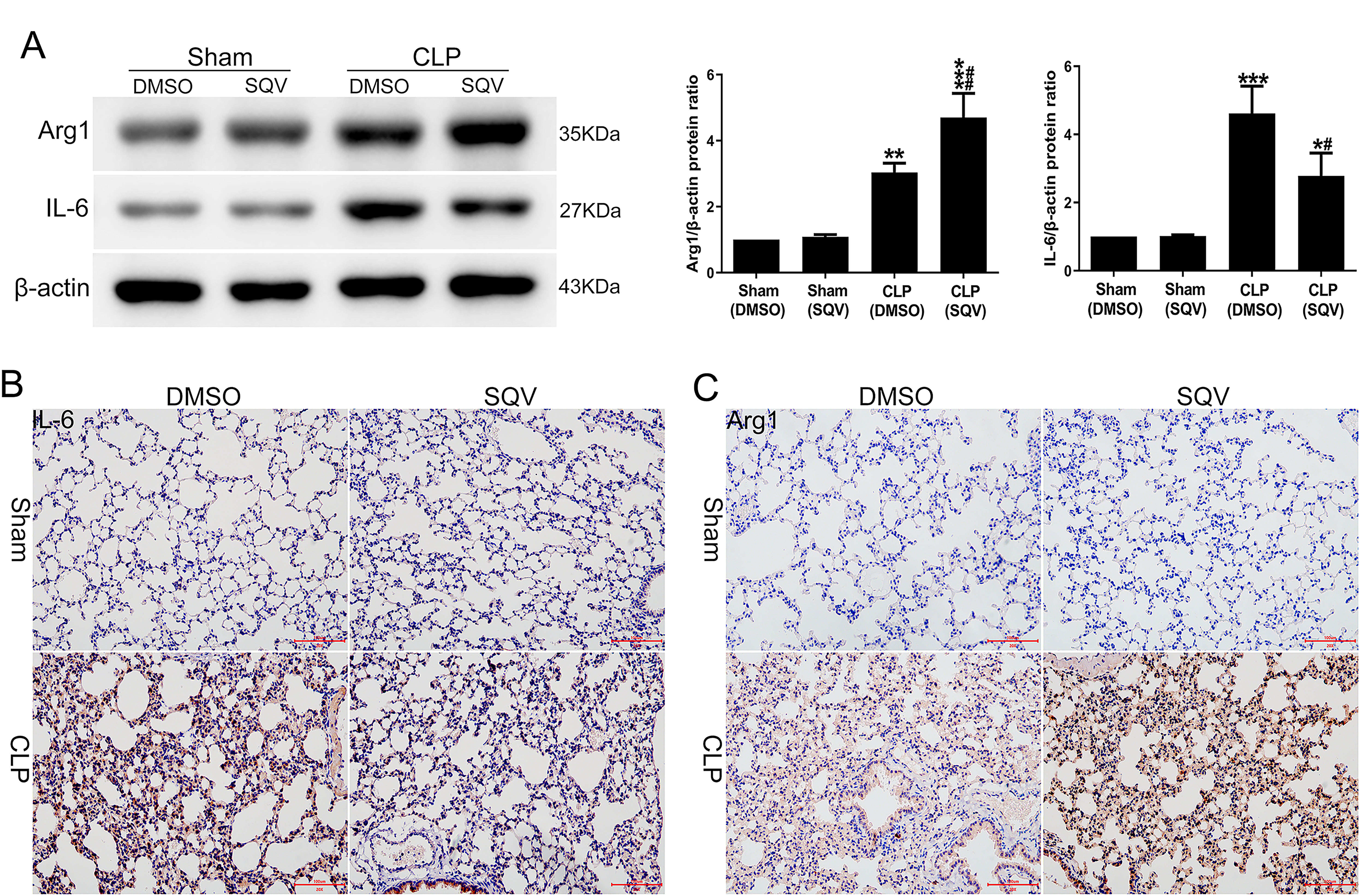

Supplement: Supplementary file 4 — Supplementary Figure 3 [file 41419_2020_3320_MOESM4_ESM.tif]

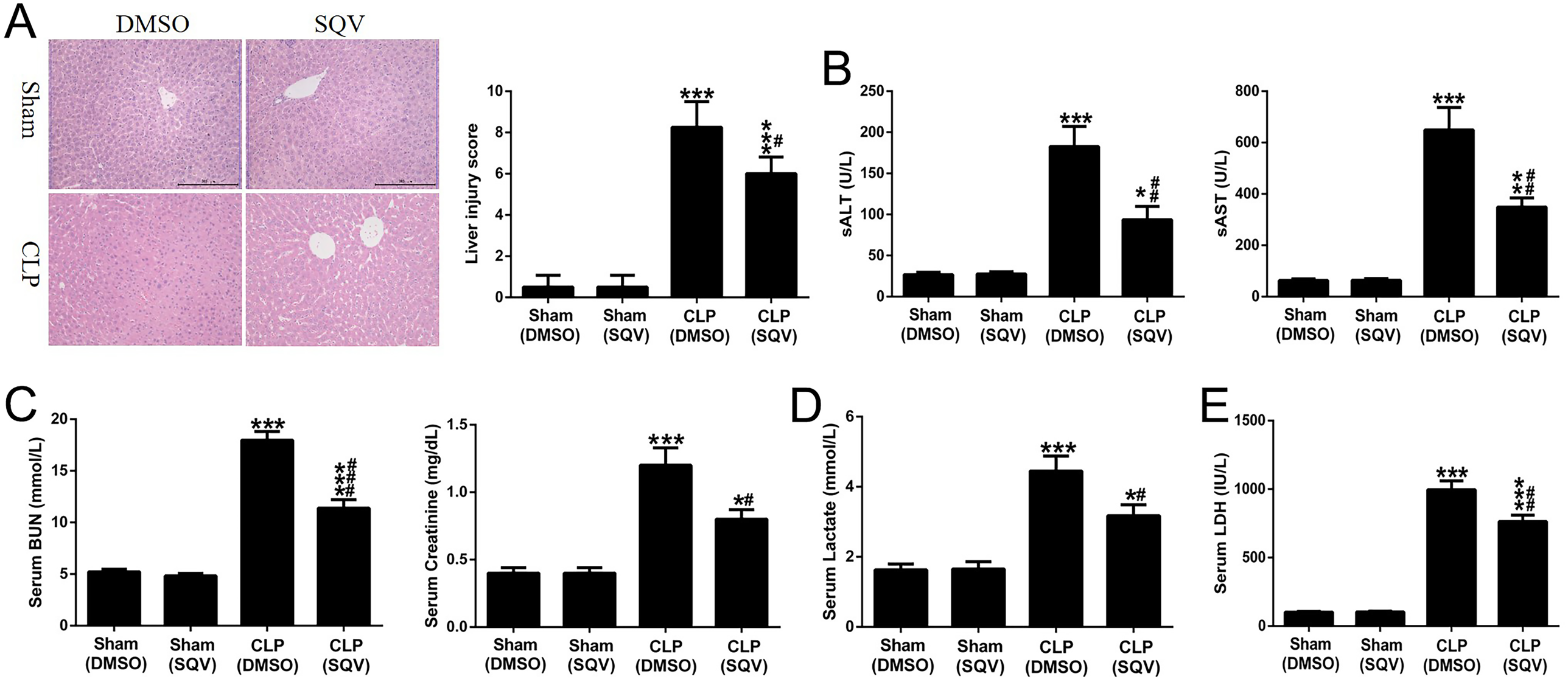

Supplement: Supplementary file 5 — Supplementary Figure 4 [file 41419_2020_3320_MOESM5_ESM.tif]
