# Supplementary material for: The HIV protease inhibitor Saquinavir attenuates sepsis-induced acute lung injury and promotes M2 macrophage polarization via targeting matrix metalloproteinase-9
Source: Cell Death Dis. 2021 Jan 11;12(1):67. doi: 10.1038/s41419-020-03320-0 (PMC7798387; doi:10.1038/s41419-020-03320-0)
Supplement: Supplementary file 6 — Supplementary Table 1 [file 41419_2020_3320_MOESM6_ESM.docx]

**Supplementary Table 1**. Primer Sequences of mice for quantitative Polymerase Chain Reaction (5'-3')

| IL-6 | Forward | TAGTCCTTCCTACCCCAATTTCC |
| --- | --- | --- |
|  | Reverse | TTGGTCCTTAGCCACTCCTTC |
| TNF-α | Forward | CCCTCACACTCAGATCATCTTCT |
|  | Reverse | GCTACGACGTGGGCTACAG |
| IL-1β | Forward | GCAACTGTTCCTGAACTCAACT |
|  | Reverse | ATCTTTTGGGGTCCGTCAACT |
| iNOS | Forward | GTTCTCAGCCCAACAATACAAGA |
|  | Reverse | GTGGACGGGTCGATGTCAC |
| IL-10 | Forward | GCTCTTACTGACTGGCATGAG |
|  | Reverse | CGCAGCTCTAGGAGCATGTG |
| Arg1 | Forward | CTCCAAGCCAAAGTCCTTAGAG |
|  | Reverse | AGGAGCTGTCATTAGGGACATC |
| Fizz1 | Forward | CCAATCCAGCTAACTATCCCTCC |
|  | Reverse | ACCCAGTAGCAGTCATCCCA |
| Mrc1 | Forward | CTCTGTTCAGCTATTGGACGC |
|  | Reverse | CGGAATTTCTGGGATTCAGCTTC |
| MMP-9 | Forward | CTGGACAGCCAGACACTAAAG |
|  | Reverse | CTCGCGGCAAGTCTTCAGAG |
