# Supplementary material for: The HIV protease inhibitor Saquinavir attenuates sepsis-induced acute lung injury and promotes M2 macrophage polarization via targeting matrix metalloproteinase-9
Source: Cell Death Dis. 2021 Jan 11;12(1):67. doi: 10.1038/s41419-020-03320-0 (PMC7798387; doi:10.1038/s41419-020-03320-0)
Supplement: Supplementary file 7 — Supplementary Table 2 [file 41419_2020_3320_MOESM7_ESM.docx]

**Supplementary Table 2.** Primer Sequences of human for quantitative Polymerase Chain Reaction (5'-3')

| MMP-9 | Forward | TGTACCGCTATGGTTACACTCG |
| --- | --- | --- |
|  | Reverse | GGCAGGGACAGTTGCTTCT |
| IL-6 | Forward | ACTCACCTCTTCAGAACGAATTG |
|  | Reverse | CCATCTTTGGAAGGTTCAGGTTG |
| TNF-α | Forward | CCTCTCTCTAATCAGCCCTCTG |
|  | Reverse | GAGGACCTGGGAGTAGATGAG |
| IL-1β | Forward | AGCTACGAATCTCCGACCAC |
|  | Reverse | CGTTATCCCATGTGTCGAAGAA |
| Arg1 | Forward | GTGGAAACTTGCATGGACAAC |
|  | Reverse | AATCCTGGCACATCGGGAATC |
| Mrc1 | Forward | TCCGGGTGCTGTTCTCCTA |
|  | Reverse | CCAGTCTGTTTTTGATGGCACT |
| Fizz1 | Forward | CCGTCCTCTTGCCTCCTTC |
|  | Reverse | CTTTTGACACTAGCACACGAGA |
